# Presynaptic regulation of the inhibitory transmission by GluR5-containing kainate receptors in spinal substantia gelatinosa

**DOI:** 10.1186/1744-8069-2-29

**Published:** 2006-09-01

**Authors:** Hui Xu, Long-Jun Wu, Ming-Gao Zhao, Hiroki Toyoda, Kunjumon I Vadakkan, Yongheng Jia, Raphael Pinaud, Min Zhuo

**Affiliations:** 1 Department of Physiology, Faculty of Medicine, University of Toronto, University of Toronto Centre for the Study of Pain, 1 King's College Circle, Toronto, Ontario M5S 1A8, Canada

## Abstract

GluR5-containing kainate receptors (KARs) are known to be involved in nociceptive transmission. Our previous work has shown that the activation of presynaptic KARs regulates GABAergic and glycinergic synaptic transmission in cultured dorsal horn neurons. However, the role of GluR5-containing KARs in the modulation of inhibitory transmission in the spinal substantia gelatinosa (SG) in slices remains unknown. In the present study, pharmacological, electrophysiological and genetic methods were used to show that presynaptic GluR5 KARs are involved in the modulation of inhibitory transmission in the SG of spinal slices *in vitro*. The GluR5 selective agonist, ATPA, facilitated the frequency but not amplitude of spontaneous inhibitory postsynaptic currents (sIPSCs) in SG neurons. ATPA increased sIPSC frequency in all neurons with different firing patterns as delayed, tonic, initial and single spike patterns. The frequency of either GABAergic or glycinergic sIPSCs was significantly increased by ATPA. ATPA could also induce inward currents in all SG neurons recorded. The frequency, but not amplitude, of action potential-independent miniature IPSCs (mIPSCs) was also facilitated by ATPA in a concentration-dependent manner. However, the effect of ATPA on the frequency of either sIPSCs or mIPSCs was abolished in *GluR5*^-/- ^mice. Deletion of the *GluR5 *subunit gene had no effect on the frequency or amplitude of mIPSCs in SG neurons. However, GluR5 antagonist LY293558 reversibly inhibited sIPSC and mIPSC frequencies in spinal SG neurons. Taken together, these results suggest that GluR5 KARs, which may be located at presynaptic terminals, contribute to the modulation of inhibitory transmission in the SG. GluR5-containing KARs are thus important for spinal sensory transmission/modulation in the spinal cord.

## Background

Fast excitatory glutamatergic synaptic transmission in the brain involves alpha-amino-3-hydroxy-5-methylisoxazole-4-propionic acid (AMPA), N-methyl-D-aspartate (NMDA), and kainate (KA) receptors. Compared with AMPA and NMDA receptors, the functions and physiological roles of KA receptors (KARs) have been discovered recently with the discovery of selective pharmacological tools [1] and the use of KA receptor subunit knockout mice [2,3]. KARs are composed of homomeric and heteromeric associations of five cloned subunits: GluR5-7, KA1 and KA2 [4]. Among KAR subunits, GluR5-7 homomers are functional kainate gated ion channels [5,6]. KA1 and KA2 form functional channels as heteromers [6,7].

KARs are present at both postsynaptic and presynaptic locations [8-13]. Generally, postysynaptic KARs mediate a small portion of excitatory synaptic transmission, whereas the presynaptic KARs regulate either glutamatergic or GABAergic transmission [11,13,14]. The modulation of γ-aminobutyric acid (GABA) release by presynaptic GluR5 KARs has been well reported in the hippocampus and cortex [2,15-22]. In the spinal dorsal horn, postsynaptic KARs mediate excitatory synaptic responses by only high intensity stimulation[14], while presynaptic KARs biphasically regulate both the excitatory [23,24] and inhibitory transmission [25,26]. The deletion of *GluR5 *abolished KAR function in cultured DRG neurons, whereas presynaptic modulation of inhibitory transmission was preserved in cultured dorsal horn neurons [26]. Thus, both GluR5 and GluR6 may regulate presynaptic GABA and glycine release in cultured spinal dorsal horn neurons. However, it is still unknown whether similar modulation exists in the substantia gelatinosa (SG) of the intact spinal slices and which KAR subunits are involved in the modulation in inhibitory transmission in this region.

Superficial lamina of the spinal dorsal horn, particularly the SG, receives nociceptive information from fine myelinated A_δ_- and unmyelinated C-primary afferent fibers [27,28]. The SG contains high densities of inhibitory interneurons with glycine, GABA and their receptors [29]. Previous work showed that KARs expression was not particular obvious in the spinal cord, with the methods of in situ hybridization [30] or immunocytochemistry [31]. However, several studies have provided persuasive evidence for functional KARs in spinal neurons [14,24-26]. A recent report showed that GluR5 is expressed in GABAergic terminals in the superficial dorsal horn [32]. Also it was suggested that functional presynaptic GluR5-containing KAR bidirectionally modulate the excitatory synaptic transmission at C-fiber afferent synapses in the SG, while GluR6-KARs inhibit glutamatergic synaptic transmission at Aδ- and C-fiber afferent synapses in the SG [23]. However, it is still unclear about the functional modulation of inhibitory synaptic transmission by KARs in the SG in spinal cord slices. In the present study, we used a GluR5-selective KAR agonist, antagonist and *GluR5*^-/- ^to show that GluR5 KARs are involved in the modulation of inhibitory transmission in the SG of spinal slices.

## Methods

### Animals

All adult C57BL/6 mice were purchased from Charles River. *GluR5*^-/- ^mice were gifts from Dr. Stephen Heinemann (Salk Institute, San Diego, CA)[2,3]. *GluR5*^-/- ^mice were maintained on a mixed 129Sv × C57BL/6 background and wild-type littermates were used as controls. All mice were maintained on a 12 h light/dark cycle with food and water provided *ad libitum*. All protocols used were approved by the Animal Care and Use Committee at the University of Toronto and conform to the NIH guidelines.

### Histochemistry

For histological processing, we used a total of 3 wild-type and 3 *GluR5*^-/- ^animals. All animals were anesthetized with an overdose of sodium pentobarbital and perfused transcardially with 20 ml of 0.1 M phosphate buffered saline (PBS; pH = 7.4) followed by cold 4% paraformaldehyde solution in PBS. Brains were then dissected out and cryoprotected in a 30% sucrose solution until sunk down. Brains were then included in embedding medium (Tissue-Tek; Sakura Finetek, Torrance, CA), fast-frozen in dry-ice, cut coronally on a cryostat (30 μm) and thaw-mounted on glass slides. Sections were then allowed to dry overnight.

We used a standard Nissl staining protocol to evaluate general anatomical features of both wild-type and *GluR5*^-/- ^animals. Briefly, brain sections were first dehydrated in a standard series of alcohols (50, 70, 95 and 100%; 2 mins each). Next, tissue was re-hydrated by incubation in alcohol solutions of decreasing concentrations (100, 95, 70, 50%; 2 mins each) and placed in distilled water for 5 mins. This step was followed by incubation of sections in a filtered solution containing 0.5% Cresyl violet in distilled water, where they remained for approximately 5 mins. Subsequently, sections were dehydrated in a series of alcohols, defatted in xylenes and coverslipped.

#### Confocal imaging

In order to determine the morphology of neurons in the SG of the dorsal horn, patch pipettes were filled with 0.1% lucifer yellow. Loaded cells were imaged using a confocal microscope (Olympus Fluoview FV1000) after the whole-cell recording; composite images were obtained by stacking optical sections into a single two-dimensional image.

### Whole-cell patch clamp recordings in spinal cord slices

Young mice (postnatal 14–34 days) were anesthetized with isoflurane. Transverse slices of the lumbar spinal cord (300 μm) were prepared as described [33]. Briefly, slices were incubated in a solution containing (mM): NaCl 95, KCl 1.8, KH_2_PO_4 _1.2, CaCl_2 _0.5, MgSO_4 _7, NaHCO_3 _26, glucose 15 and sucrose 50, and was oxygenated with 95% O_2_-5% CO_2_; pH7.4, osmolality 310–320 mOsm at 30°C for 20 mins, and then were shifted to the solution above at room temperature (21–25°C) for 30 mins to recover. A single slice was transferred to a recording chamber on the stage of a BX51W1 microscope equipped with infrared DIC optics for patch clamp recordings with an Axon 200B amplifier (Axon Instruments, CA), and continuously superfused with oxygenated recording solution at 3 ml/min. The recording solution was identical to the incubation solution except for (mM): NaCl 127, CaCl_2 _2.4, MgSO_4 _1.3 and sucrose 0. Experiments were conducted at room temperature.

Spinal lamina II could be identified as a translucent band capping the dorsal part of the gray matter under the microscope. The resting membrane potential was measured immediately after establishing the whole-cell configuration. Only neurons that had an apparent resting membrane potential more negative than -50 mV were investigated further. Depolarizing (20 – 160 pA in 20 pA steps) current injections of 0.8 s duration were applied to determine the firing pattern from resting membrane potential. Recording electrodes (2–5 MΩ) contained a pipette solution composed of (in mM): K-gluconate 120, NaCl 5, MgCl_2 _1, EGTA 0.5, Mg-ATP, 2, Na_3_GTP 0.1, HEPES 10, pH 7.2, 280–300 mOsm. Cs-MeSO_3 _was replaced by K-gluconate when inhibitory postsynaptic currents (IPSCs) were recorded. Spontaneous inhibitory postsynaptic currents (sIPSCs) were recorded in the presence of NMDA receptor-antagonist AP5 (50 μM) at the holding voltage of +10 mV. To study the relationship between the responsiveness of theGluR5 selective KAR agonist (*RS*)-2-amino-3-(3-hydroxy-5-*tert*-butylisoxazol-4-yl) propanoic acid (ATPA) and firing patterns, sIPSCs were recorded in the presence of AP5 (50 μM) and AMPA receptor antagonist GYKI53655 (50 μM) at the holding voltage of -70 mV and KCl was used as internal solution. Inward currents were also recorded in the presence of AP5 (50 μM) and GYKI53655 (50 μM) at the holding voltage of -70 mV. Access resistance was 15–35 MΩ and was monitored throughout the experiment. No correction for liquid potential was made. Recorded currents were filtered at 1 kHz and digitized at 10 kHz.

### Chemicals and drugs

All chemicals and drugs were obtained from Sigma (St. Louis, MO), except for ATPA, and QX-314, which were from Tocris Cookson (Ellisville, MO).

### Data analysis

Results are expressed as means ± SEM. Statistical comparisons were performed using the Student *t*-test or the paired t test. χ^2 ^test was used to test the significance of in the proportion of each type of neuronal firing patterns between wild-type and *GluR5*^-/- ^mice. Analysis of mIPSCs was performed with cumulative probability plots and was compared using the Kolmogorov-Smirnov (K-S) test for significant differences. The level of significance was set at *P *< 0.05.

## Results

### Spinal cord morphology in wild-type and GluR5^-/- ^mice

**Figure 1:**
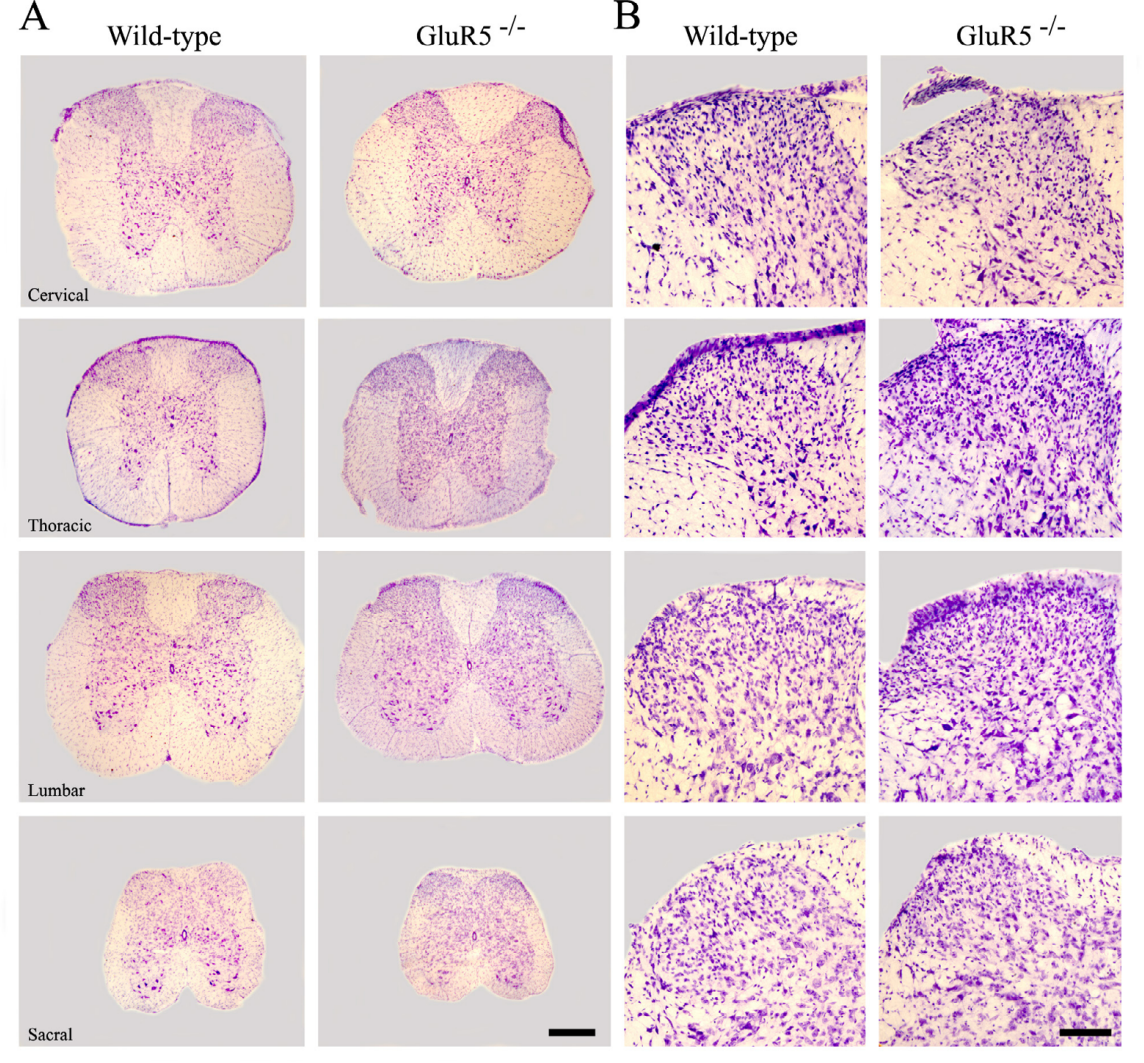
**Morphology of the spinal cord in wild-type and *GluR5*^-/- ^mice**. (A) Nissl-stained coronal sections through the spinal cord of a representative wild-type (left) and *GluR5*^-/- ^(right) mice. No obvious anatomical difference between these two strains was detected. Scale bar: 1 mm. (B) Nissl-stained sections depicting the substantia gelatinosa of wild-type (left) and knock-out (right) animals. No gross anatomical difference was detected when comparing both groups. Scale bar: 200 μm.

We investigated whether the spinal cord of *GluR5*^-/- ^animals, especially the SG, exhibited any gross abnormalities when compared to wild-type animals. Nissl stained sections of lumbar spinal cord of control and knock-out animals were used to study the gross and microscopic anatomy. Examination revealed no obvious differences in the gross anatomical organization of spinal cord between *GluR5*^-/- ^and wild-type animals (Fig. 1).

### Firing patterns of spinal SG neurons

**Figure 2:**
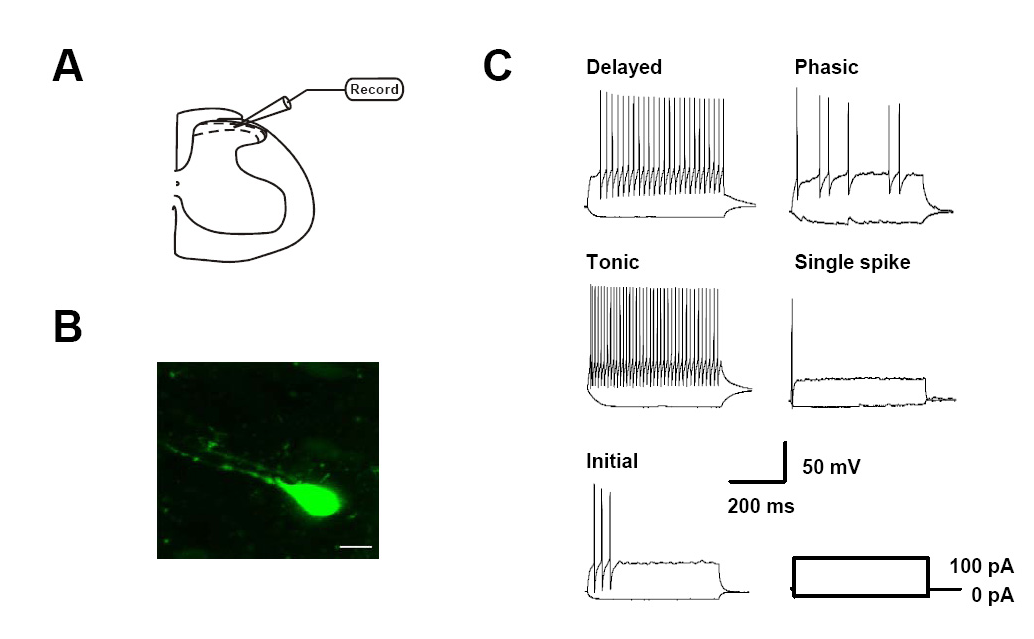
**Firing patterns in SG neurons in wild-type and *GluR5*^-/- ^mice**. (A) Diagram indicating the placement of recording electrodes in spinal dorsal horn slices. (B) Confocal image of a SG neuron in a spinal slice loaded with 0.1% lucifer yellow. Scale bar: 8 μM. (C) Firing patterns of SG neurons in wild-type mice. When injected with current steps from -20 pA to +100 pA in 800 ms (right bottom), neurons were displaying delayed firing, tonic firing, initial bursting, phasic bursting, and single spike patterns.

Dorsal horn laminae I and II neurons can be classified into five types according to their different firing patterns: tonic firing, delayed-firing, single-spike, initial-bursting and phasic-bursting neurons in rats [33]. Besides five firing patterns mentioned above, gap firing pattern was observed in lamina II neurons in dorsal horn in mice [34]. To study the firing patterns of SG neurons in both wild-type and *GluR5*^-/- ^*mice*, we performed whole-cell patch clamp recordings in visually identified wild-type (n = 43) and *GluR5*^-/- ^(n = 17) SG neurons in lamina II of spinal slices (Fig. 2A). Lucifer yellow (0.1%) was injected via the recording pipette to show recording neurons in lamina II (Fig. 2B). In wild-type mice, we found tonic firing (n = 19), delayed-firing (n = 16), single-spike (n = 4), initial-bursting (n = 3) and phasic-bursting (n = 1) patterns among 43 SG neurons (Fig. 2C). In *GluR5*^-/- ^mice, delayed-firing (n = 9), tonic firing (n = 6), single-spike (n = 1) and phasic-bursting (n = 1) patterns among 17 SG neurons (Fig. 2C). Initial-bursting pattern was not identified in *GluR5*^-/- ^mice. There was no difference between wild-type or *GluR5*^-/- ^mice in the proportion of each type of neuronal firing patterns in the total neurons recorded (*P *> 0.05). We also compared the membrane passive and active properties of neurons from wild-type and *GluR5*^-/- ^mice. No significant difference was revealed between wild-type and *GluR5*^-/- ^mice in membrane potential (-61.2 ± 1.0 mV, n = 43 versus -58.4 ± 1.5 mV, n = 17, *P *> 0.05), membrane resistance (960.1 ± 111.6 MΩ, n = 21 versus 814.7 ± 64.4 MΩ, n = 13, *P *> 0.05), membrane capacitance (35.1 ± 3.3 pF, n = 21 versus 45.9 ± 3.8 pF, n = 13, *P *> 0.05), after hyperpolarization (AHP) depth (26.9 ± 1.1 mV, n = 17 versus 22.5 ± 2.4 mV, n = 13, *P *> 0.05) or action potential threshold (-32 ± 1 mV, n = 22 versus -29 ± 2 mV, n = 17, *P *> 0.05, Table 1).

**Table 1:** Passive and active properties of SG neurons

	Wild-type	*GluR5*^-/-^
RP (mV)	-61.2 ± 1.0 (n = 43)	-58.4 ± 1.5 (n = 17)
Rm (MΩ)	960.1 ± 111.6 (n = 21)	814.7 ± 64.4 (n = 13)
Cm (pF)	35.1 ± 3.3 (n = 21)	45.9 ± 3.8 (n = 13)
AHP depth (mV)	26.9 ± 1.1 (n = 17)	22.5 ± 2.4 (n = 13)
AP threshold (mV)	-32 ± 1 (n = 22)	-29 ± 2 (n = 17)

RP: resting potential; Rm: membrane resistance; Cm: membrane capacitance; AHP: afterhyperpolarization; AP: action potential

### Activation of GluR5 increases spontaneous inhibitory transmission in SG neurons

**Figure 3:**
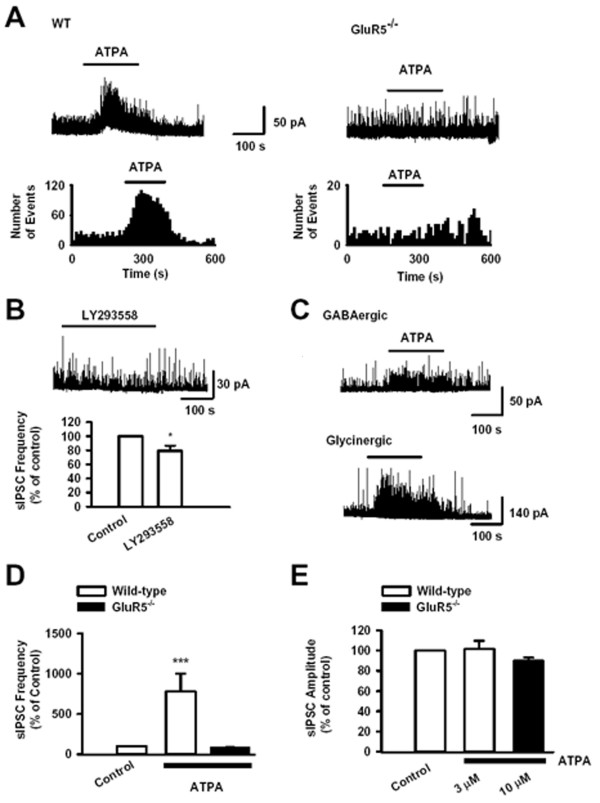
**Effect of the GluR5 agonist ATPA on sIPSCs in SG neurons**. (A) Facilitatory effect of ATPA (3 μM) on sIPSCs in one SG neuron from wild-type mouse (left panel). Effect of ATPA (10 μM) on sIPSCs (top right trace) in one SG neuron from *GluR5*^-/- ^mouse (right panel). (B) Inhibitory effect of GluR5 antagonist LY293558 (30 μM) on sIPSCs in one SG neuron from wild-type mouse (n = 9). * *P *< 0.05. (C) Effect of ATPA (3 μM) on GABAergic sIPSCs in the presence of strychnine (0.5 μM) (top trace) and glycinergic sIPSCs in the presence of bicuculline (10 μM) (bottom trace). (D) Pooled data of the effect of ATPA on sIPSC frequency from wild-type (n = 9) and *GluR5*^-/- ^(n = 5) neurons. *** *P *< 0.001 compared with the control value. (E) Pooled data of the effect of ATPA on sIPSC amplitude from wild-type (n = 9) and *GluR5*^-/- ^(n = 5) neurons.

Our previous work has shown the modulation of inhibitory synaptic transmission by presynaptic GluR5 in cultured spinal dorsal horn neurons [25,26]. To further demonstrate that the activation of KARs can regulate inhibitory transmission in the SG, we examined the effect of the GluR5 selective KAR agonist ATPA on sIPSCs *in vitro*. Recordings were made in the presence of AP5 (50 μM) to block NMDA receptors at a holding potential of +10 mV. Bath application of ATPA (3 μM) for 3 – 5 min significantly increased the frequency of sIPSCs to 484.1 ± 76.5% of control (n = 9, *P *< 0.001; Figs. 3A and 3D) in neurons tested from wild-type mice. However, the amplitude of sIPSCs was not affected in the presence of ATPA (101.8 ± 8.0 % of control, n = 9, *P *> 0.05) (Fig. 3E). In SG neurons from *GluR5*^-/- ^mice, there was no difference in the frequency of sIPSCs compared with that of wide-type SG neurons (*GluR5*^-/-^: 0.8 ± 0.1 Hz, n = 5; wide-type: 1.3 ± 0.3 Hz, n = 8, *P *> 0.05). ATPA (10 μM) had no effect on either frequency (88.4 ± 9.0%, n = 5) or amplitude (89.8 ± 3.4%, n = 5) of sIPSCs, (Fig. 3A and 3D). These results suggest that ATPA significantly facilitated the inhibitory transmission in lamina II of the dorsal horn via the activation of GluR5-containing KARs in SG neurons.

To investigate whether presynaptic GluR5 KARs are activated by endogenous glutamate, we examined the effect of bath application of GluR5 antagonist, LY293558, on sIPSCs in spinal SG neurons. LY293558 (30 μM) reversibly decreased sIPSCs frequency from 0.7 ± 0.1 Hz to 0.5 ± 0.1 Hz (n = 9, *P *< 0.05, Fig. 3B). This indicates that endogenous glutamate tonically modulates the activity of nearby inhibitory synapses via GluR5 containing KARs.

### Both GABAergic and glycinergic release were enhanced by GluR5 activation in SG neurons

Both GABAergic and glycinergic inhibitory transmissions are present in the spinal cord. Glycine and GABA are co-packaged in and co-released from interneurons in the spinal cord [35-38]. We next tested whether ATPA has selective effects on glycinergic and/or GABAergic sIPSCs. Bicuculline (10 μM) was bath applied to distinguish glycinergic component of sIPSCs, and strychnine (0.5 μM) was bath applied to separate the GABAergic component of sIPSCs [25]. We found that ATPA (3 μM) significantly increased the frequency of both glycinergic sIPSCs from 0.9 ± 0.3 Hz to 6.1 ± 1.6 Hz (n = 7, *p *< 0.05), and the frequency of GABAergic sIPSCs from 0.4 ± 0.1 Hz to 3.7 ± 0.5 Hz (n = 11, *p *< 0.05; Fig. 3C and 3D) in neurons tested from wild-type mice.

### Effect of ATPA on sIPSCs and firing patterns of spinal SG neurons

**Figure 4:**
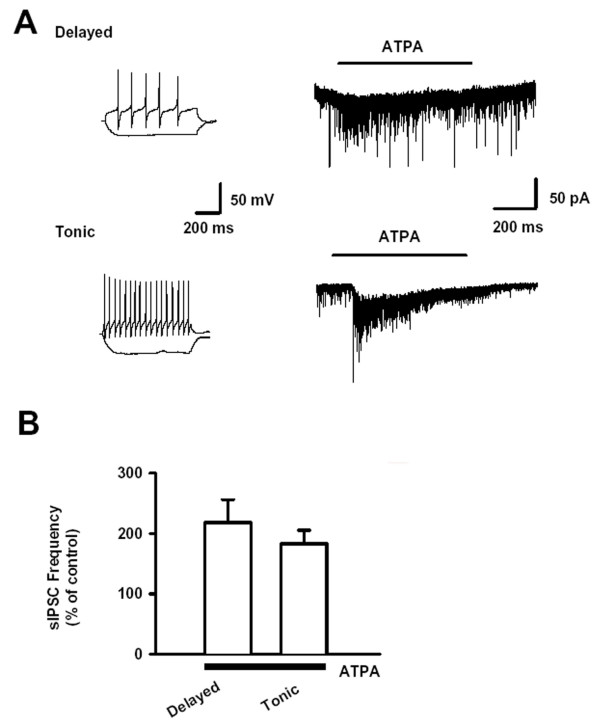
**Effect of ATPA on sIPSCs in SG neurons with different firing patterns**. (A) A typical trace showing the facilitatory effect of ATPA (3 μM) on one SG neuron with delayed firing patterns (upper trace); a typical trace showing the facilitatory effect of ATPA on one SG neuron with delayed firing patterns (bottom trace). (B) Pooled data of the effect of ATPA on sIPSC frequency in neurons with between delayed (n = 7) and tonic (n = 4) firing patterns.

To study the relationship between firing patterns and ATPA responsiveness in spinal SG neurons, inward sIPSCs were recorded in the presence of AP5 (50 μM) and GYKI53655 (50 μM) with KCl based internal solution at the holding voltage of -70 mV. ATPA (3 μM) increased sIPSC frequency in all neurons with different firing patterns as delayed, tonic, initial and single spike patterns. The percentages of ATPA's effect in neurons with two common patterns (delayed and tonic patterns) were compared. There were no significant difference between ATPA's responsiveness in neurons with these two firing patterns (218.3 ± 37.8%, n = 7 versus 182.7 ± 22.6%, n = 4, *P *> 0.05, Fig. 4).

Since most SG neurons are believed to be local interneurons [29], we wanted to know whether ATPA could induce current which may underlie the modulation of inhibitory neurotransmission. In the presence of AP5 (50 μM), strychnine (0.5 μM) and bicuculline (10 μM), inward currents could be observed in all SG neurons recorded during the application of ATPA (3 μM) (47.1 ± 14.5 pA, n = 5). The result suggests that GluR5-containing KARs exist in somatodendritic sites in spinal SG.

### Activation of presynaptic GluR5 increases the frequency of mIPSCs in SG neurons

**Figure 5:**
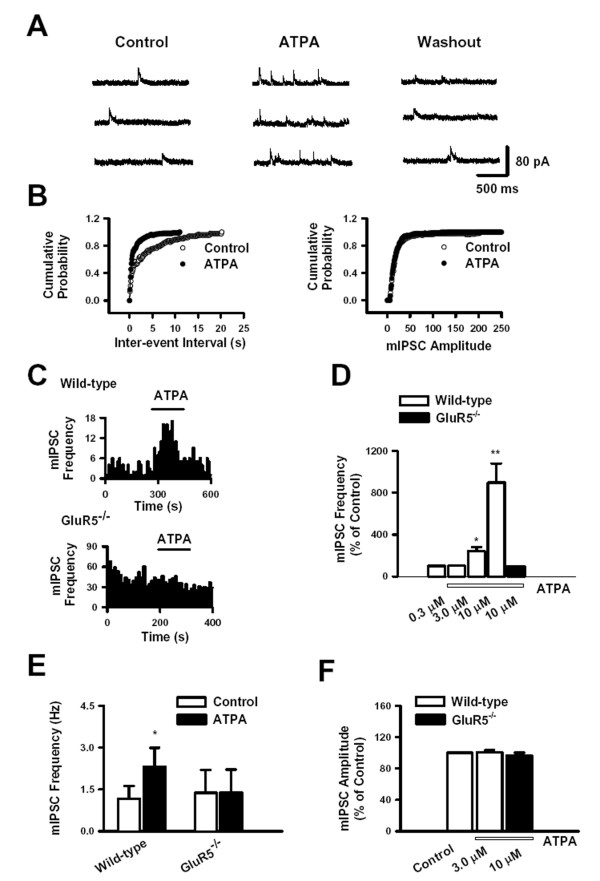
**Effect of ATPA on mIPSCs in SG neurons**. (A) Effect of ATPA (3 μM) on mIPSC frequency. From left to right, traces of mIPSCs were shown before, during and after application of ATPA (3 μM) in one wild-type neuron. (B) Cumulative histograms of mIPSC inter-event interval and amplitude before and during the application of ATPA (3 μM). (C) Typical time course of the effect of ATPAon mIPSC frequency in neurons from wild-type and *GluR5*^-/- ^mice. (D) The facilitatory effect of ATPA on mIPSC frequency was concentration dependent (0. 3 μM, n = 5; 3 μM, n = 10; 10 μM, n = 4) * P < 0.05 and ** P < 0.01. (E) Pooled data of the effect of ATPA on mIPSC amplitude in wide-type and *GluR5*^-/- ^mice. (F) Inhibitory effect of LY293558 (30 μM) on mIPSCs in one SG neuron from wild-type mouse (n = 5). * *P *< 0.05.

To further analyze the mechanism by which KARs modulate the inhibitory transmission in SG neurons, we examined the effect of ATPA on miniature IPSCs (mIPSCs). Recordings were made in the presence of TTX (0.5 μM) and AP5 (50 μM) at a holding potential of +10 mV. ATPA (3 μM) increased the frequency of mIPSCs from 1.2 ± 0.5 Hz to 2.1 ± 0.6 Hz (241.9 ± 40.2%, n = 9, *P *< 0.05, Fig. 5) in neurons tested from wild-type mice. Moreover, ATPA (0.3 – 10 μM) increased the frequency of mIPSCs in a concentration-dependent manner (Fig. 5D). However, there was no effect of ATPA on the amplitude of mIPSCs (16.2 ± 1.7 pA versus 16.1 ± 1.7 pA, n = 9, *P *> 0.05, Fig. 5F). In *GluR5*^-/- ^mice, the frequency of mIPSCs in the presence of ATPA (10 μM) was 98.4 ± 3.3% of the control (n = 5, *P *> 0.05, Fig. 5C–E). The amplitude of mIPSCs in the absence and presence of ATPA (10 μM) was 28.3 ± 5.3 pA and 27.3 ± 5.5 pA (n = 5, *P *> 0.05, Fig. 5E).

We further examined the effect of LY293558 on mIPSCs in spinal SG neurons. LY293558 (30 μM) reversibly decreased sIPSCs frequency from 0.5 ± 0.1 Hz to 0.4 ± 0.1 Hz (n = 5, *P *< 0.05, Fig. 5F). This indicates that endogenous glutamate tonically modulates the activity of nearby inhibitory synapses via presynaptic GluR5 containing KARs.

### No difference in mIPSC frequency and amplitude in SG neurons between wild-type and GluR5^-/- ^mice

**Figure 6:**
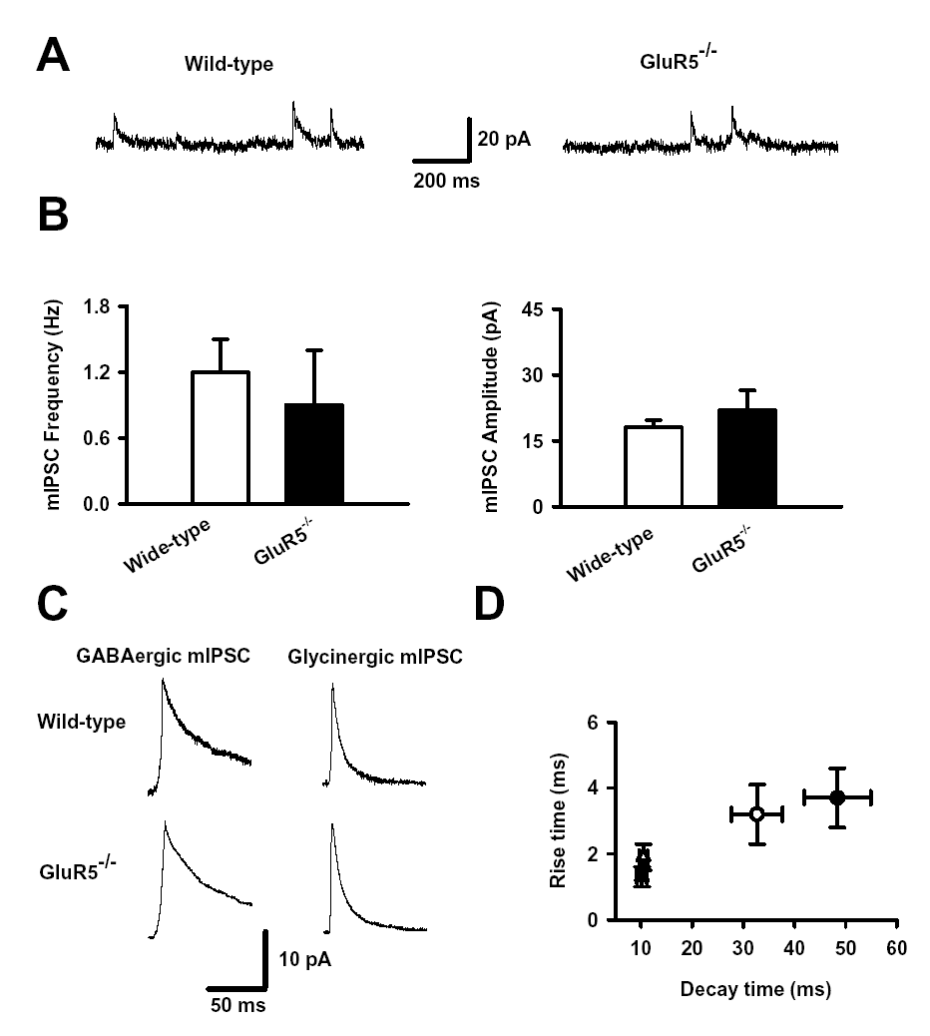
**No difference in mIPSCs in SG neurons from wild-type and *GluR5*^-/- ^mice**. (A) Typical traces of mIPSCs in SG neurons. (B) Pooled data of mIPSC frequency (left) and amplitude (right) in SG neurons. (C) Typical traces of GABAergic and glycinergic mIPSCs in two SG neurons of wild-type and *GluR5*^-/- ^mice. (D) Pooled data of decay time and rise time of GABAergic (triangle) and glycinergic (circle) mIPSCs in SG neurons from wild-type (white) and *GluR5*^-/- ^(black) mice.

As the activation of GluR5 facilitated presynaptic GABA/Glycine release, it is conceivable that the knockout of GluR5 may affect inhibitory synaptic transmission. To test this possibility, we compared mIPSCs between wild-type and *GluR5*^-/- ^mice. Surprisingly, our results showed that there was no difference in the frequency, amplitude and kinetics of mIPSCs. The frequency of mIPSCs in SG neurons from wild-type and *GluR5*^-/- ^was 1.2 ± 0.3 Hz (n = 17) and 0.9 ± 0.5 Hz (n = 8, *P *> 0.05) respectively. The amplitude of mIPSCs in wild-type and *GluR5*^-/- ^were 18.5 ± 1.6 pA (n = 17) and 22.1 ± 4.5 pA (n = 8, *P *> 0.05) respectively. Both the frequency and amplitude of mIPSCs were not significantly different compared with those of wild-type mice (Fig. 6A and 6B). In wild-type and *GluR5*^-/- ^mice, the 37–90% rise time of GABAergic mIPSCs were 3.3 ± 1.0 ms (n = 14) and 3.7 ± 0.9 ms (n = 8), the 90–37% decay time of GABAergic mIPSCs were 33.8 ± 5.0 ms (n = 14) and 48.4 ± 6.5 ms (n = 8); glycinergic mIPSCs were 2.3 ± 0.4 ms (n = 14) and 1.3 ± 0.3 ms (n = 8), the 90–37% decay time of glycinergic mIPSCs were 10.0 ± 0.8 ms (n = 14) and 10.2 ± 1.0 ms (n = 8). There were also no differences in the rise time and decay time constants of mIPSCs between wild-type and *GluR5*^-/- ^mice (*P *> 0.05, Fig. 6C and 6D). These results indicated that genetic deletion of GluR5 KARs might have no effect on the basal synaptic transmission in SG neurons.

## Discussion

Presynaptic KARs regulate GABA/glycine release in spinal dorsal horn culture [25,39]. However, it is unknown whether the similar modulation occurs in spinal cord slices. We focused here on the GluR5 modulation of inhibitory transmission in spinal cord lamina II, a region rich in interneurons and primary afferents [29]. Activation of presynaptic GluR5 by ATPA facilitates action potential-dependent and independent GABA/glycine release. *GluR5*^-/- ^mice showed normal spinal cord morphology and cellular firing properties of SG neurons as compared to wild-type mice. However, the modulation of inhibitory synaptic transmission by ATPA was abolished in *GluR5*^-/-^. Furthermore, GluR5 antagonist LY293558 inhibited both sIPSC and mIPSC frequencies in spinal SG neurons.

### Mechanism for GluR5 modulation of inhibitory transmission in SG neurons

Regulation of GABA release by KARs has been intensively studied in recent years [11,13]. The mechanisms for this modulation, however, remain controversial. For example, different research groups have reported that KAR activation can be inhibitory, facilitatory, or have no effect on mIPSC frequency [2,20,40,41]. These conflicting results likely reside in the fact that they were obtained using different preparations, dissimilar types of synapse and different pharmacological agent concentrations. However, the enhancement of sIPSC frequency by kainate or ATPA was reported in most studies, suggesting that activation of KARs could fire interneurons and thereby facilitating action potential-dependent inhibitory transmission [2,40,41].

Based on our previous work [25,26], we studied the modulation of GluR5 on inhibitory synaptic transmission in the SG region of slices. The expression of GluR5 in the dorsal horn is still a matter of debate. While one study reported a large number of primary afferents expressing GluR5-7 in the dorsal horn [42], another study showed low levels of GluR5 expression [43]. Another recent report showed a low level of co-localization between GluR5 and GAD65-immunopositive terminals in the adult rat [32]. We found that the activation of GluR5 by ATPA increased the frequency of sIPSCs in all SG neurons tested. However, the amplitude of sIPSCs was not altered by ATPA. Furthermore, ATPA increased the frequency but not amplitude of mIPSCs. These results indicate that: (1) ATPA activation increased both action potential-dependent and independent inhibitory synaptic transmission in the SG region. In favor of this notion, we also noticed that the effect of ATPA on the frequency of sIPSCs was more than that of mIPSCs. Furthermore, ATPA could also induce inward currents in spinal SG neurons, which indicates that GluR5 KARs are located at somatodendrtic sites in SG in spinal cord. (2) The increase of mIPSC frequency indicates a change in the probability of inhibitory neurotransmitter release by activation of presynaptic terminals. However, more work is needed to address whether voltage-dependent calcium channels are involved in this modulation, similar to what is reported in cultured dorsal horn neurons.

### Modulation of both GABAergic and glycinergic inhibitory transmission by GluR5

Both GABA and glycine mediate inhibitory transmission in the dorsal horn. Moreover, they both exist in and are co-released from the same synaptic vesicles in dorsal horn interneurons [35-38]. Therefore, the co-localization of GABA and GluR5 may also reflect, to some extent, the co-localization of glycine and GluR5. Consistently, we found that ATPA markedly increased the frequency of sIPSCs in all SG neurons tested in the presence of bicuculline or strychnine, suggesting that the activation of GluR5 facilitates both GABAergic and glycinergic transmission in the SG.

### Tonic activation of GluR5 KARs modulate inhibitory synaptic transmission in SG neurons

Previous studies have shown that endogenous activation of presynaptic GluR5 KARs in interneurons modulate the inhibitory transmission in hippocampus, basolateral amygdala and spinal cord [25,41,44]. Therefore, we wanted to know whether tonic activation of presynaptic GluR5 KARs modulates inhibitory synaptic transmission in SG neurons in the spinal cord. To address this question, we tested the effect of GluR5 antagonist, LY293558 on inhibitory synaptic transmission. Our results showed that LY 293558 could decrease the frequency of both sIPSCs and mIPSCs. The result suggests the tonic activation of GluR5 in the spinal SG. However, when compared the frequency and amplitude of mIPSCs in SG neurons between wild-type and *GluR5*^-/- ^mice, we found that the frequency and amplitude of mIPSCs were not significantly different from each other. The results from pharmacological data and from *GluR5*^-/- ^mice, therefore, seem different. The discrepancy may be due to developmental compensation in basal inhibitory synaptic transmission in the knockout mice. Further studies are needed to elucidate this question.

### Pathophysiological role for the modulation of GABA and glycine release by GluR5

KARs were suggested to be involved in pathophysiological functions such as epilepsy, fear memory and chronic pain [45-48]. Our previous results in dorsal horn slices found that the KAR-mediated current can only be elicited upon the stimulation of the afferent axon at an intensity strong enough to activate high threshold Aδ and C fibers, suggesting the critical role of spinal dorsal horn KARs in nociception[14]. Behavioral studies using pharmacological and genetic tools show the involvement of KARs, in particular GluR5, in both acute nociception and chronic pain [46,49-51]. Considering the wide expression of functional KARs from the DRG, the spinal cord and supraspinal structures such as the anterior cingulate cortex [52,53], the exact functional sites for KARs are largely unknown.

Our results demonstrate that presynaptic GluR5 in lamina II of the spinal dorsal horn plays a significant role in the regulation GABA and glycine release. Regulation of inhibitory transmission in the dorsal horn is essential for nociceptive processing and other sensory transmission [54,55]. Since activation of presynaptic GluR5 in the SG decreased the postsynaptic interneuronal excitability, the net effect may cause an increase in excitability, thereby enhancing the nociceptive transmission. Therefore, the blockade of GluR5 at the spinal level would have an analgesic effect. Accordingly, intrathecal injection of a selective GluR5 antagonist reduced nociceptive responses to CFA inflammation and GluR5 expression was increased in the spinal cords of CFA treated animals [50]. Moreover, *GluR5*^-/- ^mice showed reduced behavioral responses to inflammatory pain compared to wild-type mice [46]. Taken together with previous results, the present study suggests that spinal GluR5 may play an important role in pathological pain.
